# Identifying goals, roles and tasks of extended scope physiotherapy in Dutch primary care- an exploratory, qualitative multi-step study

**DOI:** 10.1186/s12913-020-05986-w

**Published:** 2021-01-06

**Authors:** Ferdinand Bastiaens, Di-Janne Barten, Cindy Veenhof

**Affiliations:** 1 Physical Therapy Sciences, Program in Clinical Health Sciences, University Medical Center Utrecht, Utrecht, The Netherlands; 2 Research Group Innovation of Human Movement Care, University of Applied Sciences Utrecht, Utrecht, Netherlands; 3 Department of Rehabilitation, Nursing Sciences and Sport, University Medical Center Utrecht, Utrecht, Netherlands

**Keywords:** Physical therapy modalities, Extended scope, Multi-step design, Primary health care

## Abstract

**Background:**

Rising healthcare costs, an increasing general practitioner shortage and an aging population have made healthcare organization transformation a priority. To meet these challenges, traditional roles of non-medical members have been reconsidered. Within the domain of physiotherapy, there has been significant interest in Extended Scope Physiotherapy (ESP). Although studies have focused on the perceptions of different stakeholders in relation to ESP, there is a large variety in the interpretation of ESP. Aim: To identify a paradigm of ESP incorporating goals, roles and tasks, to provide a consistent approach for the implementation of ESP in primary care.

**Methods:**

An exploratory, qualitative multi-step design was used containing a scoping review, focus groups and semi-structured interviews. The study population consisted of patients, physiotherapists, general practitioners and indirect stakeholders such as lecturers, health insurers and policymakers related to primary care physiotherapy. The main topics discussed in the focus groups and semi-structured interviews were the goals, skills and roles affiliated with ESP. The ‘framework’ method, developed by Ritchie & Spencer, was used as analytical approach to refine the framework.

**Results:**

Two focus groups and twelve semi-structured interviews were conducted to explore stakeholder perspectives on ESP in Dutch primary care. A total of 11 physiotherapists, six general practitioners, five patients and four indirect stakeholders participated in the study. There was a lot of support for ‘decreasing healthcare costs’, ‘tackling increased health demand’ and ‘improving healthcare effectiveness’ as main goals of ESP. The most agreement was reached on ‘triaging’, ‘referring to specialists’ and ‘ordering diagnostic imaging’ as tasks fitting for ESP. Most stakeholders also supported ‘working in a multidisciplinary team’, ‘working as a consultant’ and ‘an ESP role separated from a physiotherapist role’ as roles of ESP.

**Conclusions:**

Based on the scoping review, focus groups and interviews with direct and indirect stakeholders, it appears that there is sufficient support for ESP in the Netherlands. This study provides a clear presentation of how ESP can be conceptualized in primary care. A pilot focused on determining the feasibility of ESP in Dutch primary care will be the next step.

## Background

High-quality primary healthcare is an important priority for Western societies. This ambition is threatened from two sides. On the one hand, the demand for healthcare in a primary care setting has increased due to an aging population and an increase in the number of chronically ill patients [1]. On the other hand, general practitioners (GPs) in primary care face increasing workloads while the average of weekly work hours remains the same. Both developments put pressure on sustaining the quality of primary healthcare and have made primary care organization transformation a priority in several countries [1–4].

One of the ways these challenges in (primary) healthcare have been met, is to reconsider the roles of non-medical members of the healthcare team and substitute tasks traditionally carried out by physicians [5]. By these new ‘Extended Scope’ roles, healthcare providers aim to increase patient satisfaction and improve access to care with comparable or better quality and efficacy at lower healthcare costs [6, 7]. With respect to the domain of physiotherapy, there has been significant interest in Extended Scope Physiotherapy (ESP) over the last 20 years within healthcare systems of the United Kingdom, Canada and Australia [7–11]. Especially in settings providing services to patients with musculoskeletal disorders, physiotherapists have emerged as key providers in such new redistributed roles. For example, initiatives for the treatment of patients with common musculoskeletal disorders have been implemented in emergency departments, orthopaedic clinics and the primary care setting [12–14]. Research suggests that extended scope physiotherapists achieve similar or better results in musculoskeletal complaints regarding diagnostic accuracy, effectiveness of care, care utilization and cost of care compared to GPs [15].

Although ESP has widely been reported in literature, there is a large variety in the interpretation of ESP between the countries and settings in which ESP is implemented. Remarkably, most of these studies focus on ESP in hospital based settings. A clear representation of how ESP could be conceptualized specifically in primary care is currently lacking.

This representation is necessary, because the context of a primary care setting is substantially different from the context of secondary care in which ESP can be implemented. For example, external stakeholders play an important role in primary care. Increased collaboration, development opportunities and a shared understanding between stakeholders are required for the extended scope role to flourish [16]. Currently, there is insufficient insight into perceived legitimacy from relevant stakeholder groups in primary care, which is based on the value of, the confidence in and the boundaries of the extended scope role [17].

The three most important stakeholder groups regarding ESP in primary care are patients, physiotherapists and GPs. Several studies have already been performed regarding patient perceptions on ESP. A qualitative study concerning patient perceptions of the ESP role showed themes that were important regarding the quality of service: provision of information, professional skills, interpersonal skills, outcome, and patient care pathway [13]. A survey which focused on ESP in primary care showed that patients supported the intended new roles of the ESPs regarding the treatments of patients with musculoskeletal disorders [14].

A qualitative study on the perspectives of physiotherapists on working as an ESP in an orthopaedic outpatient clinic concluded that the physiotherapists experienced that, although the job can be stressful, it is also very satisfying [18]. Furthermore, a survey of physiotherapists and physiotherapy employers on clinical specialization and extended scope showed participants are supportive of the roles of the clinical specialists and advanced practitioners within the profession [19]. To our knowledge, no studies focused on GP perspectives on ESP yet.

Although several studies have focused on the perceptions of different stakeholder groups in relation to ESP, no clear interpretation of ESP in primary care exists. This is either due to perceptions of stakeholder groups being focused on ESP in hospital based settings or perceptions of stakeholder groups being absent.

Therefore, we aim to identify a stakeholder supported paradigm of ESP, incorporating goals, roles and tasks, to provide a consistent approach for the implementation of ESP in primary care through collaboration with patients, physiotherapists, GPs and other stakeholders in primary care. In order to make this paradigm applicable to clinical practice in primary care, it is best to be captured in a framework format.

## Methods

### Design

**Fig. 1:**
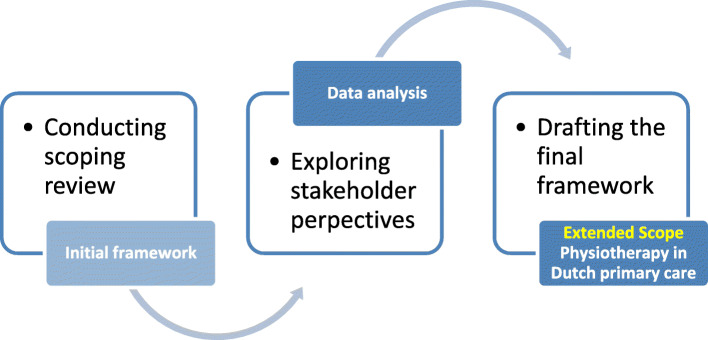
Iterative process of developing the framework of Extended Scope Physiotherapy in Dutch primary care

An exploratory, qualitative multi-step design was used, based on the iterative process used by Harding et al. and Bravo et al. in order to complete the ESP framework [20, 21]. The iterative process of the multi-step design is illustrated in Fig. 1. This multi-step approach includes a scoping review, focus groups and the drafting of a final framework.

### Scoping literature review on ESP

The first phase is to conduct a scoping review in order to develop an initial framework of ESP in primary care. This scoping review follows the scoping review model portrayed in Arksey and O’Malley and the proposed recommendations found in Levac et al. [22, 23] and consists of the following steps:

#### Identifying the research question

The aim of the scoping review was to identify an initial framework of ESP in primary care, which could be used as a foundation to explore Dutch stakeholder perspectives on ESP. The following research question was formulated for the scoping review: “What characteristics or aspects are featured in the paradigm of Extended Scope Physiotherapy in primary care?”

#### Identifying relevant studies

A search strategy was set up with the aid of an information specialist and consisted of keywords and subject headings. Four databases were used to systematically search articles (Medline, Embase, Cinahl and Sportdiscus) relevant to the topic of ESP. The search string was built upon a combination of the professions (e.g. physiotherapy), domain (e.g. extended scope), and outcomes (e.g. decision making). The complete search string can be found in Appendix 1. The search was conducted in 2017 and updated in July 2019. Studies found through the search results were imported and managed in Rayyan QCRI [24].

#### Study selection

Studies containing a definition of, or criteria for ESP in a primary care setting were included. Since the scope of physiotherapy differs between countries, studies describing a scope-extending feature in the originating country were included as well. There was no limitation regarding design or year of publication when selecting the articles. Articles that used definitions adopted from other studies were excluded. Articles written in a language different from English or Dutch were excluded as well.

Study selection was performed by subsequent steps. First, all imported studies were scanned for duplicates. To determine eligibility, the studies were subsequently scanned on title and abstract. This was followed by checking for full text availability and the screening of full text articles. The selection process was conducted by the first author (FB).

#### Charting the data

To provide an overview of the included studies, the following data were extracted using a standardized extraction form: first author; year of publication; study design; ESP description, definition and/or criteria.

#### Collating, summarizing, and reporting results

The analytical approach used to create the initial framework of ESP in Dutch primary care was the ‘framework’ method developed by Ritchie & Spencer [25]. The first step was familiarization of the collected data by gathering ESP descriptions, definitions and criteria from the included studies. Secondly, all key themes were identified in order to further develop the framework. Thirdly, data were indexed in textual form by coding the relevant information from the studies. Fourthly, data were classified according to the relevant part of the thematic framework. Finally, the identified themes were mapped using tables and diagrams. This initial framework was visualized by creating a mind map consisting of the codes and identified themes which were present among multiple studies.

### Exploring stakeholder perspectives

The initial framework was further discussed on clinical relevance for primary care by a range of direct and indirect stakeholders of ESP in Dutch primary care. The stakeholder perspectives were analyzed in order to index the data and to identify themes. These themes were then used to adjust the initial framework in order to make the paradigm fitting for Dutch primary care.

#### Participants

Physiotherapists working in primary care settings were approached to participate in this study. They were invited via general newsletters, social media posts and personal invitations based on work-related connections. In addition, recruitment focused on lecturers and internship supervisors from the Physiotherapy Bachelor of the University of applied Sciences Utrecht, as well as Master students and alumni of Physiotherapy Science, Program in Clinical Health Sciences, Utrecht University. The recruitment strategy of GPs initially focused on primary care settings nationwide. Patients were contacted via physiotherapists working in primary care practices in and around Utrecht.

Subsequently, perspectives of several indirect stakeholders regarding ESP, like policymakers, financiers and lecturers on the domain of primary care, were gathered. Healthcare departments of several insurance companies were approached as financial stakeholders. Lecturers from both General Practice and Physiotherapy programs were contacted as educational stakeholders, respectively related to the University Medical Center Utrecht and the Utrecht University of Applied Sciences. Professional associations from both GPs and physiotherapists were contacted as well. Policymakers that we contacted were the Ministry of Health, Welfare and Sport, a physiotherapy accreditor organization and the Dutch Extended Scope Society.

Participants were included if they were ≥ 18 years and able to speak the Dutch language. In addition, physiotherapists and GPs had to be involved with the primary care setting during their participation of the study. Patients had to have experience with the treatment of musculoskeletal complaints in the primary care setting. No exclusion criteria were used in this study. In order to get a clear view of the different perspectives of the stakeholder groups, homogeneity was preferred in the forming of the groups [26].

#### Procedure

Physiotherapists and GPs were invited to participate into separate focus groups. With respect to feasibility, GPs were invited to take part in an online focus group by way of FocusgroupIT (www.focusgroupit.com) instead of a face-to-face focus group. The aimed number of participants for the focus groups was between 6 and 12 persons per group [27]. The primary researcher (FB) led the focus group discussions.

The views of patients and indirect stakeholders on ESP were gathered by semi-structured interviews. Those interviews were held at a location of their choice or by telephone. Voice recording was used during both the focus group sessions and the semi-structured interviews.

#### Data collection

Prior to the focus groups and semi-structured interviews, ‘age’, ‘sex’ and ‘familiarity with ESP’ of the participants were noted. Additionally, ‘work experience’ and ‘postgraduate degree’ was gathered from physiotherapists and GP’s. ‘Level of education’ and ‘type of health problem’ was collected from patients and ‘professional discipline/area of specialisation’ was noted in indirect stakeholders.

An interview guide was developed based on the initial framework. Major topics reflected the broader themes found in the scoping review. Within these topics, sample questions were formulated, specifically focussing on subcomponents of the themes. The interview guide developed for patients was limited to topics related to patient-experienced features of ESP. The full topic list is presented in Appendix 2. Prior to the interview, participants were given an explanation about ESP in the information letter. If necessary, the themes and their sub-components were explained during the interview. In order to get the participants’ full perspective on ESP, they were asked on their view of every single theme (e.g. every goal, task and role) in the initial framework.

#### Data analysis

Once again, the analytical approach by Ritchie & Spencer was used to refine the initial framework of ESP in Dutch primary care [25]. The method involves the initial framework as a working analytical framework that is used to index the data, whilst remaining sufficiently flexible to allow the incorporation of additional themes. The process of familiarization of the collected data, identification of themes and indexing of the data in by coding was used on the transcripts. The classification and mapping of the data served to refine the mind map of the initial framework. This process is in accordance with approaches to establish rigor in qualitative research, particularly in establishing credibility, which represents means of granted value to qualitative findings [28].

### Drawing final framework

The final framework of ESP was drafted by the researchers, capturing the themes adopted by direct and indirect stakeholders. NVivo software was used to aid the analysis and generation of additional themes. Analyses were performed by the primary researcher (FB) and a member check was performed by another researcher (JB).

### Ethical considerations

Ethics approval was received from the Medical Ethics Committee of the University Medical Center Utrecht (18–137/C). Participants received the participant information letter and an informed consent form by e-mail from the primary researcher prior to their participation. A reminder was sent a few days before the start of the study. Written informed consent was obtained from all participants prior to participation in the study.

## Results

### Literature review

#### Study selection & data chart

**Fig. 2:**
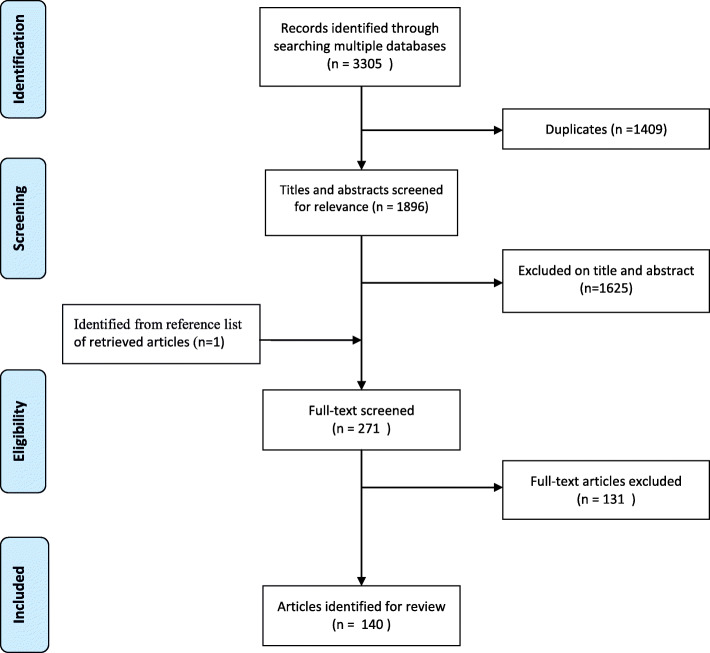
Study selection flow chart

In total, 1896 unique entries were identified through the literature search of the databases. After screening the title and abstract for inclusion, 270 studies were selected for full text screening. One study was added through a search of the articles reference list. In total, 140 studies were included in this scoping review on identifying characteristics or aspects concerned with ESP in primary care (Fig. 2). Included articles were qualitative, quantitative or descriptive in nature and contained experimental designs, literature reviews, convention abstracts. A complete overview of the included studies can be found in Appendix 3.

#### Initial framework

**Fig. 3:**
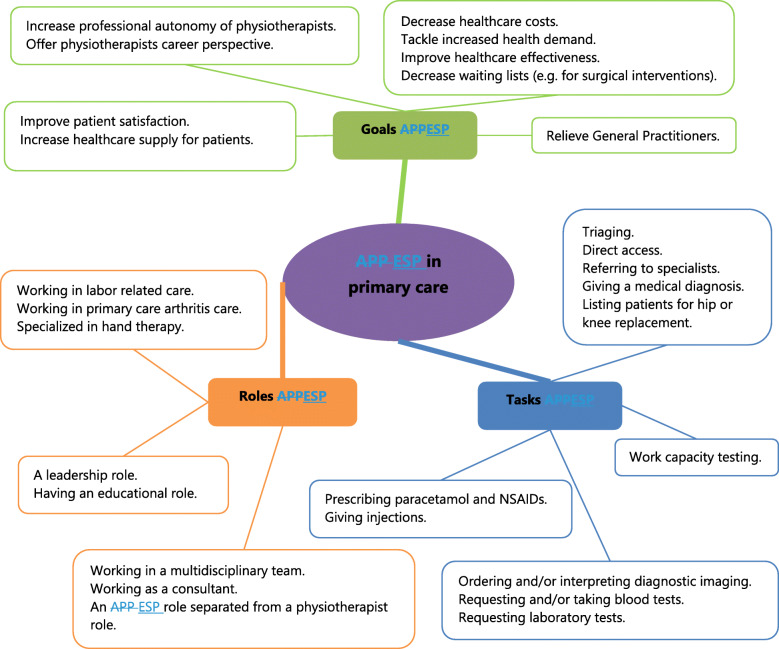
Initial framework Extended Scope Physiotherapy (ESP) in Dutch primary care

Based on the included studies by the scoping review, an initial framework was created containing goals, roles and tasks associated with ESP in primary care (Fig. 3). Themes encompassing desired results for improving existing structures or tackling problems were linked to Goals of ESP. Themes describing concrete activities were linked to Tasks of ESP, whereas broader conditions related to job position or behavior were linked to Roles of ESP. *Goals* of ESP found in the literature focused on tackling major trends in healthcare (e.g. decreasing healthcare costs, improving healthcare effectiveness or decreasing waiting lists) [29–31]. Furthermore, goals specified one or more groups that ought to benefit from ESP, such as increasing autonomy for physiotherapists, relieving GP’s or increasing patient’s access to care [14, 32, 33]. Described *tasks* of ESP varied both by country and by time period. A total of 31 and 20 articles described triaging and referring to specialists as ESP tasks respectively [34, 35]. Articles focused on prescribing NSAIDs or ordering diagnostic imaging were numerous as well, with 28 and 31 descriptions respectively [36, 37]. Descriptions of ESP-roles sometimes focused on a specific setting, such as hand therapy or arthritis care [38, 39]. More general descriptions, such as consultants or part of a multidisciplinary team were also found in the literature [40, 41].

### Stakeholder perspectives

#### Participants

Two focus groups and twelve semi-structured interviews were conducted to explore stakeholders perspectives on ESP in Dutch primary care. One focus group contained nine physiotherapists (6 males, median age: 29 years, familiarity with ESP: *n* = 3) and one online focus group contained six GPs (5 males, median age: 40 years, familiarity with ESP: *n* = 0). Two physiotherapists (2 males, median age: 44 years, familiarity with ESP: *n* = 1) were interviewed additionally to enlarge the chance of saturation. Furthermore, five patients (2 males, median age: 53 years, familiarity with ESP: n = 0) and four indirect stakeholders (Policy officer, Lecturer, Healthcare buyer, Chairman professional organization, 4 males, median age: 37 years, familiarity with ESP: n = 3) were interviewed.

#### Perspectives on extended scope physiotherapy

The initial framework presented in Fig. 2 was the starting point for discussions with stakeholders in focus groups and interviews. Their perspectives regarding the goals, tasks and goals of ESP are summarized below. An extended summary of examples illustrating contributions of the stakeholders in narrative form are presented in Appendix 4.

##### Goals of ESP

In general, participants experienced difficulties in envisioning clear goals for ESP. Nevertheless, there was a noticeable difference in the support of the different potential goals as extracted by the scoping review.

Regardless the different stakeholders, there was a lot of support for ‘decreasing healthcare costs’, ‘tackling increased health demand’ and ‘improving healthcare effectiveness’ as important goals. A majority also supported ‘relieving GPs’. However, some participants questioned whether the addition of ESP would have that effect. By way of illustration, a physiotherapist mentioned: *“So, what we are already doing a bit is to take out that musculoskeletal group in particular. A nurse practitioner also tackles the easier conditions. But the result is that the GP, who hoped for a milder consultation, actually saw an increase in the consultation hour.” (physiotherapist, age range 40–49 years).*

Moreover, most participants viewed ‘improving patient satisfaction’, ‘increasing professional autonomy of physiotherapists’ and ‘offering physiotherapists career perspective’ as potential positive effects rather than goals. Little support was given to ‘decreasing waiting lists’ and ‘increasing healthcare supply’ for patients, because the goals were deemed irrelevant to the Dutch healthcare system.

##### Tasks of extended scope physiotherapists

Physiotherapists tended to be more willing to assign tasks to ESP than GPs. However, most agreement was reached on ‘triaging’ and ‘referring to specialists’ as tasks fitting for ESP. This agreement is illustrated by a GP who indicated: “*As far as I am concerned, estimations and differential diagnostics in the musculoskeletal area could be useful.” (GP, age range 30–39 years).*

GPs were divided on ‘requesting diagnostic imaging’, but there was agreement in favor of the task among the other stakeholders. In contrast, only little support was shown for ‘interpreting diagnostic imaging’. While ‘direct access’ and ‘work capacity testing’ were supported, most stakeholders did not see it as tasks specifically related to ESP. ‘Listing patients for hip or knee replacement’ was not supported by GPs, patients and indirect stakeholders, illustrated by the participating lecturer: “*Yes, I think this goes pretty far too. If you are going to do that, then you do not need orthopedics. The question is whether you should want that. When you need orthopedics, they have to give that judgment. And then the orthopedic surgeon will provide surgical care. You can say: I refer to the secondary care.”(Lecturer, age range 30–39 years).*

The stakeholders expressed mixed reactions on ‘giving a medical diagnosis’, ‘requesting laboratory tests’ and ‘giving injections’. Reactions on ‘requesting blood tests’ were mixed as well, although the stakeholders generally did not support the ‘taking of blood tests’. When ‘prescribing’ was discussed, the majority of the stakeholders was in favor of prescribing paracetamol, but the prescription of NSAIDs received less support. A patient noted: “*Paracetamol, yes. Anti-inflammatory drugs I think it is tricky. I would like to have a second opinion from a doctor then.”(Patient, age range 50–59 years).*

##### Roles of extended scope physiotherapists

There was large agreement among stakeholders regarding the potential roles in ESP. Most stakeholders supported ‘working in a multidisciplinary team’, ‘working as a consultant’ and ‘an ESP role separated from a physiotherapist role’. A GP stated: *“Ideally, in collaboration with the GP and especially specialists. “(GP, age range 40–49 years).*

Additionally, the majority of the stakeholders opposed having ‘an educational role’, ‘a leadership role’ and ‘a role as doctor of physiotherapy’. An example illustrating a patient’s views on the leadership role: “*No, when I look at my own work, you have people who grow into a [leadership role]. And sometimes you do not do any work at all that you’re used to do, but you know the ropes. So yes, but you need different qualities and not every ESP could do it.”(Patient, age range 40–49 years).*

The roles ‘working in labor related care’, ‘working in primary care arthritis care’ and ‘specialized in hand therapy’ were mostly viewed as optional specializations instead of key aspects of ESP.

##### Additional themes

Additional themes also arose from the data. ‘Sufficient work experience’ was noted by all stakeholders as a requirement for ESP. A physiotherapist mentioned: “*I wonder, when you look at setting it up and dividing it in the neighborhood, if a GP is waiting for a 26-year-old ESP that takes over many of its tasks. I think that a lot of experience and age makes sense.”(Physiotherapist, age range 20–29 years).*

Physiotherapists also indicated the ‘profiling of their profession’ as an important goal related to ESP. This goal focuses more on the overarching physiotherapeutic profession in the Netherlands, whereas the already mentioned goal of offering physiotherapists’ career perspective focuses particularly on a therapists’ personal perspective. Another theme that arose was ‘ESP structured as a specialist or as a generalist’. Some participants showed interest in an ESP framework aimed at enhancing physiotherapeutic specialists in certain niches, where other participants focused more on ESP as a generalist aimed at triaging and diagnosing patients with musculoskeletal complaints in general practice. The participating policy officer viewed it as such: *“I really see an ESP as a kind of super specialist. So the moment you really start working in a part of your domain, then I think you need a good basis for that. So also be able to apply those extra skills to be able to develop well in that area.”(Policy officer, age range 30–39 years).* While both roles do not have be mutually exclusive in ESP, some participants showed concerns of ESP being set up too widely.

### Drafting the final framework

**Fig. 4:**
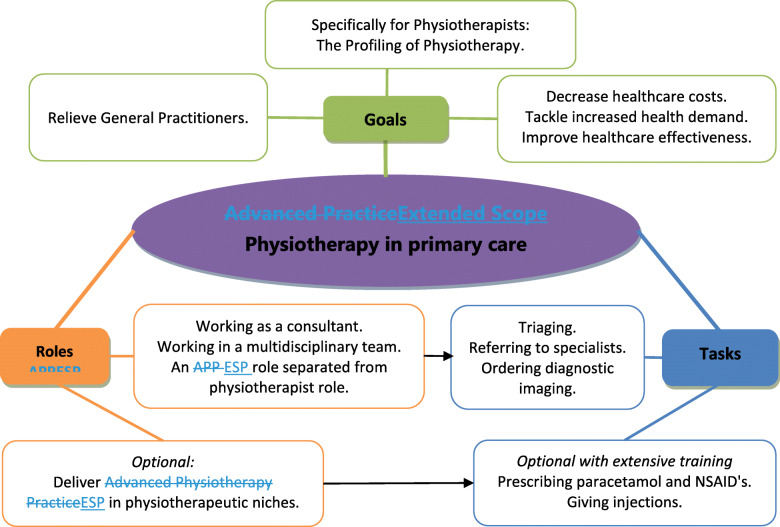
Final framework Extended Scope Physiotherapy in Dutch Primary care

Based on the identified stakeholder perspectives in the Netherlands, the initial, literature-based framework was adjusted in order to fit the framework to primary care. The goals ‘improving patient satisfaction’, ‘decreasing waiting lists’ and ‘increasing healthcare supply’ were removed from the framework, whereas ‘increasing professional autonomy of physiotherapists’ and ‘offering physiotherapists career perspective’ were replaced by ‘the profiling of Physiotherapy’. The tasks ‘direct access’, ‘work capacity testing’, ‘giving a medical diagnosis’, ‘requesting laboratory tests’ and ‘requesting and/or taking blood tests’ were removed entirely and ‘ordering and/or interpreting diagnostic imaging’ was reduced to ‘ordering diagnostic imaging’. Furthermore ‘prescribing paracetamol and NSAID’s’ and ‘giving injections’ were labeled as tasks for ESP which were optional with extensive training. In the section ‘Roles ESP’, the themes ‘working in labor related care’, ‘working in primary care arthritis care’ and ‘specialized in hand therapy’ were replaced with ‘deliver ESP in physiotherapeutic niches’, which was labeled as optional. The roles ‘an educational role’, ‘a leadership role’ and ‘a role as doctor of physiotherapy’ were removed. The final framework is illustrated in Fig. 4.

## Discussion

The purpose of this study was to identify a stakeholder supported paradigm of ESP, incorporating goals, roles and tasks, to provide a consistent approach for the implementation of ESP in primary care. Looking at the identified paradigm, the main goals of ESP are to decrease healthcare costs, to tackle increased health demand and improve healthcare effectiveness. The roles in which an ESP acts are more generic in nature, focusing on consulting and/or participating in a multidisciplinary team. The main task of an ESP will be triaging and, if necessary, referring to specialists and ordering diagnostic imaging. Additional themes in the paradigm are the possibility for ESP in physiotherapeutic niches and requirements for becoming an ESP, such as a minimal amount of work experience and additional education.

Several studies previously examined extended scope through the perspectives of different stakeholders. Wiles et al. studied the perceptions of different key stakeholders on the ESP role in Australia [42]. They found agreement on the value of ESP in improving the efficacy and efficiency of health service delivery, achieving positive patient outcomes and offering opportunities for interdisciplinary learning among colleagues. This largely corresponds with the findings in our study related to the goals of ESP in primary care. Although it was not stated as a goal of ESP, the identified support for ESP in a multidisciplinary team reflects a positive view of interdisciplinary learning. Looking at the implementation of extended scope, previously identified key themes consisted for example of proactively addressing barriers; legislative issues; developing, accrediting and delivering a curriculum supporting physiotherapists to work outside of the usual scope [43]. These themes have not been studied in our study, due to the lack of an established form of ESP. However, these are important points that need to be taken into account in expanded research on the paradigm of ESP.

Looking at contemporary reforms of the Dutch primary care, the identified paradigm provides clinical relevance on the potential role of ESP. The Dutch government aims at substitution from secondary care to primary care [44, 45]. With the increased pressure on GPs, more supporting healthcare providers are needed to relieve the GPs and, simultaneously, to maintain quality of care. Therefore, substitution is seen as a driving force to innovations in healthcare professions [46]. Substitution can also add to reducing healthcare costs, with ESP improving diagnostic accuracy and decreasing unnecessary referrals to specialists. Furthermore, the ESP goal to improve healthcare effectiveness fits in the restructuring model of Kaljouw and Van Vliet (2015), regarding personalized care [45]. This model sets up an expansion of tasks, focusing on improving one’s function instead of improving the illness.

### Strengths and limitations

One of the strengths of this study was the iterative design. This design enabled drafting the framework in a thorough manner. The literature review provided a broad foundation in which the majority of final themes were present. In addition, the diverse groups of stakeholders provided a broad spectrum of perspectives on ESP applicable to the setting of primary healthcare. Furthermore, discussions with stakeholders have helped to create support for ESP in primary care. Additionally, due to the systematic interview style and the explanations of how the tasks, roles and goals worked out in practice, participants received a clear understanding during the interview. This provided a beneficial contribution to the cohesiveness of the final framework.

Some limitations should be mentioned as well. For example, the recruitment of GPs appeared to be more difficult than expected. Their busy schedule might have played a role, or their interest in the topic of ESP. Moreover, the barrier to assemble in one location at the same time withheld participants as well. This was partly tackled by setting up the online focus group for the GPs and taking individual interviews. However, a sample bias still occurred. Polled participants who were less invested in ESP, were more eager to refuse participation. The small sample size has likely led to bias, although it is not clear in which way this bias runs. Moreover, general support for ESP has only been investigated to a limited extent due to the lack of interaction between the stakeholder groups. Although it is a strength of this study to give each stakeholder group room for their own perspective, discussions among stakeholders could have provided a more fleshed out paradigm. Furthermore, the recruitment strategy mainly focused on participants in the city of Utrecht and its metropolitan area, which is predominantly urban. Stakeholder perspectives from rural areas might provide benefits to the paradigm in future studies. Looking at the international validity of the paradigm, countries with lower levels of urban density will likely benefit from taking into account a factor such as ‘access to care’ when developing their version of ESP in primary care. The identified paradigm generally aligns with countries which have already implemented ESP in primary care. Therefore, this paradigm ought to be useful for countries with a similar primary care setting that are interested in implementing ESP as well.

### Recommendations

The framework provides a realistic and advantageous model for the development of ESP in primary care in the Netherlands. There seems to be sufficient support regarding the paradigm of ESP in view of several direct and indirect stakeholders in primary care. Therefore, it would appear that the time has come to study ESP more thoroughly by determining its’ feasibility by way of an observational pilot study. In consistence with the identified goals, tasks and roles of ESP, diagnostic accuracy and patient and GP satisfaction should be used as outcome measures in this trial. Furthermore, emphasis should be put on requirements and preconditions for physiotherapists who can be eligible for ESP. More research is also recommended on the perspectives of healthcare providers related to primary care, like general practice based nurse specialists, district nurses and specialists in secondary care, such as neurologists, orthopedic surgeons and rheumatologists. Looking at the established paradigm, these healthcare providers will most likely be influenced in their work by the introduction of ESP and therefore can be counted as direct stakeholders. Another priority in further research is studying interactive discussions between stakeholder groups in order to identify a more conscientious paradigm of ESP in primary care.

## Conclusion

This study aimed to identify a paradigm for ESP that fits to Dutch primary care based on both literature and stakeholder perspectives. Based on the scoping review, focus groups and semi-structured interviews with various direct and indirect stakeholders, it appears that there is sufficient support for ESP in the Netherlands. The main goals of ESP are to decrease healthcare costs, to tackle increased healthcare demand and to improve healthcare effectiveness. The roles in which an ESP acts are more generic in nature, focusing on consulting and/or working in a multidisciplinary team. The main task of an ESP is triaging and, if necessary, referring to specialists and ordering diagnostic imaging. An observational pilot study focusing on determining the feasibility of ESP in Dutch primary care will be the next step.

## Data Availability

Overviews of the data derived from the scoping review and interviews supporting the conclusions of this article are included within the appendices section. The complete datasets used and analyzed during the current study are available from the corresponding author on reasonable request.

## Appendix

**Table 1:** Search string scoping review

Population:	
***MeSH (Pubmed)***	***Title / abstract***
Physical therapists (mesh from 2012)	Physical therap*
Physical therapy modalities (mesh until 2012)	Physiotherap*
Musculoskeletal manipulations	Manual therap*
***Emtree (Embase)***	
Physiotherapist	
Physiotherapy	
Manipulative medicine	
***Heading (Cinahl, SPORTDiscus)***	
Physical therapists	
Physical therapy	
Manual therapy	
**Intervention:**	
***MeSH/Emtree***	***Title / abstract***
n/a	advance practi*
***Heading***	Advanced practi*
Scope of practice	Advance scope*
	Advanced scope*
	consultant physio*
	Enhanced practice*
	enhance practice*
	Enhancing practice*
	enhance scope*
	Enhanced scope*
	enhancing scope*
	Expand practice*
	Expanded practice*
	Expanding practice*
	Expand scope*
	Expanded scope*
	Expanding scope*
	Extending scope*
	extended scope*
	extended practice*
	Extending practice*
	role expan*
	role enhan*
	role exten*
	Roles expan*
	Roles enhan*
	Roles exten*
	scope of practice
	new role*
**Outcome:**	
***MeSH***	***Title / abstract***
Decision making	Outcome
Patient satisfaction	Clinical reasoning
Clinical decision making (mesh from 2016)	Patient experience*
Education	Patient preference*
***Emtree***	Substitution
Decision making	Diagnostic*
Patient satisfaction	Operational*
Clinical decision making	Organis*
Education	Organiz*
Health care organization	
***Heading***	
N/a	

The asterisk sign was used for truncation during the search

**Table 2:** Topic list focus groups

Topic	Sample questions (optional)
Goals ESP	What do you see as the goal of an ESP?
Tasks ESP	Can you give examples of tasks you think an ESP can perform?
	Do you think there are tasks that an ESP cannot perform?
Rolls ESP	Looking at the roles, which ones would you see being fulfilled by the ESP?
	Which roles do not fit with the ESP?
	Can you tell whether this changes the collaboration with others?
Supporting conditions	Which things does the ESP need to do its job well?
	Can you tell what would change in the ESP’s collaboration with others?
Education ESP	Can you tell what should be covered in the study program?
	What do you think about admission requirements for the study program? Can you think of examples?
Confidence ESP	1. Would you go to an ESP yourself with a complaint? Why?
	What would you consider when choosing an ESP or another practitioner?

**Table 3:** Overview of the included studies in the scoping review

Author (Year)	Country	Design		Goals					Tasks					Roles				Additional features
			Factors	General healthcare	Patients	GPs	Physio-therapists	Other	General	Medicinal treatment	Diagnostics	Work-related	Other	General roles	Disciplinary specific	Sector specific	Other	
Aiken (2008) [28]	Canada	inter-rater reliability study		**X**					**X**									Study was not performed in, but is applicable to primary care
Akehurst (2019)	UK	Pilot		**X**		**X**			**X**	**X**	**X**						**X**	
Allan (2017)	UK	Pilot		**X**					**X**		**X**							
Ambury (2010)	Canada	Pilot														**X**		
Atkins (2003)	UK	Qualitative design					**X**			**X**								
Bailey (2015)	USA	Clinical commentary		**X**							**X**							
Belot (2017)	Canada	Survey among physiotherapists									**X**							
Bennet (2017)	UK	Pilot		**X**	**X**				**X**	**X**	**X**				**X**			
Beswetherick (2015)	UK	Literature review, qualitative study		**X**	**X**		**X**			**X**								
Boucaut (2013)	Australia	Exploratory study										**X**				**X**		
Boyles (2011) [29]	USA	Clinical commentary		**X**					**X**		**X**							
Braund (2011)	New Zealand	Survey								**X**								
Brody (2009)	USA	Review					**X**								**X**			
Brown-Benedict (2008)	USA	Exploratory study													**X**			Post-baccalaureate level.
Bubanja (2019)	USA	Exploratory study													**X**			
Burn (2014)	UK	Pilot							**X**									
Bury (2013)	WCPT (global)	Mixed method, Exploratory study							**X**									
Caine (2016)	UK	Exploratory study		**X**		**X**			**X**	**X**	**X**							
Candy (2011)	UK	Exploratory study		**X**							**X**							
Candy (2015)	UK	Exploratory study		**X**					**X**		**X**				**X**			
Chong (2015) [31]	Canada	Survey									**X**							
Connoly (2006)	USA	Opinion piece					**X**		**X**									
Crowell (2016)	USA	Exploratory study		**X**					**X**		**X**							
Dascanio (2015)	UK	Clinical commentary					**X**						**X**					
De Sa (2014)	Portugal	Systematic review														**X**		**pediatric primary care**
Delany (2019)	Australia	Qualitative design																Ethical considerations
Desjardins (2016) [14]	Canada	Survey		**X**	**X**		**X**		**X**	**X**	**X**							
Downie (2019)	UK	Exploratory study				**X**			**X**	**X**	**X**				**X**			
Eaton (2017)	UK	News article															**X**	Master’s degree level
Ellis (2005)	UK	Delphi study									**X**				**X**			
Ellis, R (2018)	New Zealand	Exploratory study									**X**							
Fennely (2018)	Ireland	Systematic review		**X**	**X**				**X**	**X**	**X**		**X**	**X**				
Ferguson (2011)	UK	Exploratory study		**X**					**X**	**X**	**X**							
Fish (2019)	UK	Pilot							**X**							**X**		
Froment (2019)	WCPT (global)	Survey							**X**	**X**	**X**	**X**	**X**			**X**		
Gaskell (2010)	UK	Exploratory study		**X**					**X**				**X**	**X**	**X**			
Gazsi (2010)	USA	Exploratory study													**X**			
Gosling (1999)	UK	Clinical commentary					**X**											Master’s degree level
Gosling (2019)	UK	Exploratory study					**X**		**X**						**X**			
Grbin (2013)	New Zealand	Exploratory study					**X**											
Green (2008)	UK	Exploratory study					**X**							**X**	**X**			
Greenhalgh (2016)	UK	Clinimetric study		**X**					**X**									
griffiths 1 (2012)	UK	Survey		**X**						**X**	**X**							
griffiths 2 (2012)	UK	Exploratory study		**X**						**X**	**X**			**X**				
Grimmer (2017)	Australia	Literature review		**X**	**X**	**X**	**X**	**X**	**X**	**X**	**X**			**X**		**X**	**X**	
Hattam (1999) [33]	UK	Exploratory study		**X**					**X**									
Hawke (2017) [30]	USA	Exploratory study		**X**	**X**											**X**		**Satellite clinic**
Hensher (1997)	UK	Review		**X**	**X**				**X**									
Hensman-Crook (2017)	UK	Pilot		**X**	**X**	**X**			**X**	**X**	**X**				**X**			
Higginson (2017)	UK	Exploratory study		**X**	**X**				**X**	**X**								**Telephone-based**
Hill (2016)	UK	Clinical trail			**X**								**X**		**X**			
Hing (2012)	New Zealand	Clinical commentary							**X**									
Holdsworth (2008)	UK	Survey		**X**		**X**	**X**		**X**	**X**	**X**			**X**				**Casemanager Role**
Huijbregts (2007)	USA	Clinical commentary							**X**		**X**							
Humprey’s (2010)	UK	Diary/feasibility study							**X**					**X**	**X**			
Igwesi-Chidobe (2019)	UK	Qualitative study				**X**								**X**				
Innes (2015)	UK	Clinical commentary		**X**					**X**	**X**	**X**							
Jadon (2009)	UK	Exploratory study		**X**					**X**		**X**					**X**		
Johnson (2011)	UK	Exploratory study/pilot												**X**	**X**			
Johnston (2016)	Australia	Clinical commentary		**X**	**X**				**X**			**X**				**X**		
Jones (2015) [16]	UK	Qualitative study		**X**						**X**				**X**	**X**		**X**	**Research, training and peer support**
Kamimura (2007)	USA	Clinical commentary		**X**					**X**	**X**	**X**				**X**			
Kennedy (2011)	Canada	Survey							**X**		**X**					**X**		
Kerridge-Weeks (2017)	UK	Exploratory study		**X**					**X**									
Kersten (2007) [7]	UK	Systematic review		**X**		**X**	**X**											
Langridge (2015)	UK	Qualitative study					**X**				**X**							
Langridge (2019) [40]	UK	Qualitative study		**X**	**X**	**X**			**X**	**X**	**X**			**X**				
Lee (2019)	UK	Exploratory study							**X**		**X**							
Lehman (2017)	USA	Clinical commentary											**X**					**Nutritional advice**
Lineker (2011) [34]	Canada	Exploratory study			**X**				**X**	**X**						**X**		**Recommend Splints**
Lundon (2008)	Canada	Exploratory study		**X**	**X**				**X**							**X**		
Lundon (2011) [38]	Canada	Exploratory study		**X**	**X**				**X**		**X**					**X**		
Lundon (2013) (1)	Canada	Mixed method, Exploratory study												**X**		**X**		
Lundon (2013) (2)	Canada	Mixed method, exploratory study		**X**	**X**				**X**					**X**		**X**		
O Mir (2018) (1)	Ireland	Literature review		**X**												**X**		**Pediatric care**
O Mir (2018) (2)	Ireland	Exploratory study		**X**					**X**		**X**				**X**	**X**		**Pediatric care**
Mabry (2019)	USA	Exploratory study		**X**		**X**			**X**	**X**	**X**							
Maiorana (2019)	Australia	Exploratory study							**X**									
Marks (2017)	Australia	Systematic review		**X**	**X**	**X**									**X**			
Martini (2017)	UK	Pilot				**X**			**X**					**X**				
May (2017)	UK	Pilot		**X**		**X**			**X**					**X**				
Mccloy (2001)	USA	Clinical commentary											**X**					**Nutrition, mental health, stress management**
Mcilroy (2019)	UK	Survey							**X**						**X**			
Mcneily (2012)	UK	Exploratory study			**X**					**X**								
Monteith (2019)	UK	Exploratory study		**X**	**X**	**X**	**X**		**X**	**X**					**X**			
Morley (2019)	UK	Exploratory study		**X**	**X**				**X**		**X**				**X**			
Morris (2014)	Australia	Systematic review		**X**						**X**								
Morris (2017)	UK	Qualitative design		**X**					**X**	**X**								
Morris (2019)	UK	Mixed method							**X**						**X**			
Mullan (2016)	UK	Qualitative					**X**			**X**								
Murphy (2011)	Ireland	Clinical trail							**X**						**X**	**X**		
Myers (2019)	USA	Root cause analysis													**X**			**Extensive education**
Noblet (2018)	Australia	Survey (protocol)		**X**	**X**					**X**					**X**			
Norris (2002)	USA	Year end report			**X**					**X**								
O’Mahony (2015)	Ireland	Qualitative study		**X**			**X**		**X**						**X**	**X**		
Oakley (2015)	UK	Systematic lit. Review		**X**					**X**		**X**							
Oriel (2003)	USA	Survey						**X**								**X**		**school-based physiotherapy**
Palma (2017)	UK	Mixed method, Exploratory study					**X**							**X**				
Parfitt (2012)	UK	Retrospective review		**X**					**X**									
Prescott (2011)	UK	Exploratory study							**X**									**Master modules**
Quicke (2019)	UK	Pilot		**X**					**X**						**X**			
Raymer (2011)	Australia	Survey													**X**			
Razmjou (2017) [36]	Canada	Prospective study		**X**	**X**				**X**		**X**							
Rose (2009) [37]	UK	Audit		**X**							**X**				**X**	**X**		
Ross (2019)	UK	Pilot		**X**	**X**	**X**			**X**	**X**	**X**							
Rushton (2008)	Australia	Review article		**X**	**X**	**X**	**X**		**X**	**X**	**X**							
Salmon (2017)	UK	Mixed method pilot			**X**	**X**			**X**									
Samsson (2016)	Sweden	Randomized controlled trail		**X**	**X**				**X**	**X**	**X**				**X**			
Sedgeley (2016)	UK	Consensus study																**Postgraduate education**
Sephton (2010)	UK	Prospective cohort		**X**					**X**		**X**							
Shakinovski (2011)	UK	Cross-sectional		**X**							**X**							
Shoemaker (2012)	USA	Exploratory study			**X**				**X**									
Soever (2011)	Canada	Pilot		**X**	**X**				**X**		**X**							
Stamm (2011)	EULAR (Europa)	Survey		**X**					**X**							**X**		
Stanhope (2012)	Australia	Systematic review							**X**	**X**	**X**					**X**		
Stenner (2018)	UK	Systematic review		**X**						**X**								
Stevenson (2003)	UK	Clinical commentary		**X**			**X**		**X**					**X**	**X**			
Stevenson (2011)	UK	Clinical commentary		**X**										**X**	**X**			
Stevenson (2011) (2)	UK	Qualitative study		**X**										**X**	**X**			
Stigmar (2014)	Sweden	Qualitative study					**X**					**X**				**X**		
Stigmar (2015)	Sweden	Survey										**X**				**X**		**Insurance medicine education**
Stokes (2007)	UK	Clinical commentary					**X**				**X**							
Suckley (2012)	UK	Delphi study							**X**					**X**				
Thomas (2003)	USA	Survey					**X**								**X**			
Thompson, A. (2017)	UK	Case report								**X**								
Thompson, J. (2017)	UK	Systematic review		**X**	**X**										**X**			
Thomson (2017)	UK	Audit		**X**					**X**		**X**							
Unger (2005)	South Africa	Survey		**X**						**X**								**Describes situations in other countries**
Unger (2006)	South Africa	Survey								**X**								**Describes situations in other countries**
Vassilevskaja (2019)	UK	prospective analysis		**X**						**X**					**X**			
Vits (2010)	UK	Clinical commentary		**X**										**X**	**X**			
Vliet Vlieland (2015)	The Nether-lands	Clinical commentary													**X**	**X**		**Describes situations in other countries**
Walker (1999)	USA	Survey		**X**					**X**									
Wang (2007)	Taiwan	Pharmaco-logical trail											**X**					**Electrotherapy**
Warmington (2011)	Canada	Cross-sectional evaluation		**X**	**X**		**X**			**X**	**X**					**X**		
Warmington (2015)	Canada	Qualitative study			**X**					**X**	**X**					**X**		
Welsh (2014)	UK	Qualitative study		**X**								**X**						
White (2019) [35]	UK	Delphi study								**X**								
Whittaker (2019)	Canada	Clinical commentary									**X**							
Yardley (2008) [19]	Canada	Survey					**X**		**X**	**X**	**X**				**X**			

**Table 4:** Typical quotes of direct and indirect stakeholders per topic

Goals	Physiotherapist		General Practitioners	Indirect stakeholder
Decrease waiting lists	No, I do not see that as a goal. Due to the emergence of independent treatment centers and the current healthcare system, you actually see that there are no or hardly any waiting lists.		This does not currently play in my region.	When an ESP is used and prevents a patient from unnecessarily going to the orthopedist and therefore occupying the consultation hour, I think the waiting lists will be shortened.
Increase healthcare supply for patients	But what [physiotherapist] just rightly points out is that the supply is shifting. It does not change, so in principle it is not a larger supply. Instead of going to the doctor, you now go to the ESP, which basically performs the same tasks.		Patients often do not know what the best care is by the forest of healthcare providers. More care provision does not lead to better care.	I wonder if you will increase the healthcare supply. I do not think you can shed the healthcare supply, but you are trying to send insured patients directly to the right place where they can receive care.
Decrease healthcare costs	Yes, we are of course cheaper than the GP. So that certainly applies to this. I do not know if a different rate applies. If there are other training requirements, there may also be a higher rate than a physiotherapist You should see it as a specialism.		The biggest challenge of care will be that we have to do more and more for less and less money (and ensure sufficient staff working in the healthcare sector).	I certainly think that it can lead to a reduction in healthcare costs, because I am convinced that some of the patients who are referred to the second line do not actually have to be there. If you can get that percentage of people out of the front, then you reduce those healthcare costs
Tackle increased health demand	Yes, we have a lot to do with this. And we often look at patients differently than the GP. In that sense, I think that the quality is only better if we also look at it. We also have a lot of experience with the elderly, so we can also help them a lot.		Particularly in the elderly, there is a lot to be gained (therapeutic and preventive) with low-threshold access to good movement care and advice.	I think there is a place for it. It is also being said that the second-line care will disappear. Hospitals in the current form are going to disappear. This is increasingly going to the periphery. And that is precisely where that super specialist who is needed in practice and the community. You will need more of that.
Relieve General Practitioners	So what we are already doing a bit is to take out that musculoskeletal group in particular. A nurse practitioner also tackles the easier conditions. With the result that the GP, who hoped for a milder consultation, but what you actually see is an increase in the consultation hour		That may be a welcome side effect, but should not be a reason to (yet) introduce a new profession. Complaints of the musculoskeletal system are not a big burden for most GPs, and there are also many abnormalities (rheumatic, paraneoplastic and otherwise) that do not belong primarily to the physiotherapist.	I certainly see that. You also hear that the GPs are too busy. Because they are the gatekeeper, they obviously need to know something. What we hear is that there are also quite a few people with musculoskeletal complaints. We think that the physio has much more knowledge of it. So yes, if they are already taken away from the GP, then you are sure to relieve the GPs.
Increase professional autonomy of physiotherapists	I thought more with professional autonomy that you have more handles as a physio to do more things. But that you will get more opportunities for the patient outside of exercise therapy, mobilization, etc. That it is something that is more for yourself. That is indeed possible, it could make it more attractive.		Especially nice for the physiotherapist, but that is in itself insufficient reason and should not be a primary goal. We must not introduce a new medical profession “because we want it so badly”	I do not think it is an important goal, but it is a result that occurs when you have that function. But then it must be guaranteed. It cannot be the case that every physiotherapist suddenly has such a forward position. So you will demonstrably have to have knowledge and skills.
Improve healthcare effectiveness	I think that there should be a kind of shift and that this is just a nice step for a person who really sends the whole team or a neighborhood or a village and ensures that the care is more effective.		You can never be opposed to that, right?	It is an important point to put physiotherapy on the map as the professional in movement care who knows what it is about. That it will show added value in the context of sensible efficient care
Improve patient satisfaction	In my experience we do it very well with the patients, high marks. While the care is not always good, or equally efficient. So I would like to place an exclamation mark at patient satisfaction in the sense of: Let’s focus on that carefully before we get a very satisfied patient and deliver something half-baked.		If the physio does what a patient would want immediately, perhaps, but more patient satisfaction? There remains a group that wants to have the doctor’s opinion.	I think that patients might ultimately be more satisfied with care in general. That less sending from the box to the wall and just to one person who understands business. But we do not have to do anything about patient satisfaction with physiotherapy, because on average it is very high. So we do not have to do much about that, but maybe in general healthcare.
Offer physiotherapists career perspective	I graduated 3 years ago and from the group I graduated a number of them have already stopped because they no longer find it attractive. They started working in other places, in other branches. How can we keep those people in the end?		That would be a good side effect, but it would not be a primary goal.	I certainly think so. It offers new challenges, new possibilities. You will profile yourself even more as a specialist. You can put yourself down well, so it does offer perspective. Maybe not financially, but in professionalism. I think it is a bycatch.
Tasks	Physiotherapist	General Practitioners	Patients	Indirect stakeholder
Triaging	Yes, very suitable as ESP I would say. Perhaps the most important task.	As far as I am concerned, estimations and differential diagnostics in the musculoskeletal area could be useful.	I think that a physiotherapist has more knowledge of the musculoskeletal system and a GP has more general knowledge. I think it is good to take over.	Yes I think that’s fine, as long as it falls within the domain of the physiotherapist.
Prescribing paracetamol and NSAID’s	Yes, that you can do so with additional knowledge. If we indeed know when you can or cannot prescribe it. That you cannot do it in combination with other medication. Anyway, if that is in the training that makes you ESP, I can imagine it is one of the tasks.	I find the assessment of which medication goes quite far if you cannot properly interpret comorbidity	Paracetamol, yes. Anti-inflammatory drugs I find tricky. I would like to have a second opinion from a doctor then.	Yes, both are basically over the counter medicine. So whether you say that, or whether the neighbor says it, or if someone thinks that he is going to swallow painkillers. That is not really an extra task. These are freely available products in the Netherlands. That is their own responsibility. You can advise that. But if you want to prescribe it as an advice for pain management, if you are aware of the effect and dosage, I do not think that’s a problem.
Ordering and/or interpreting diagnostic imaging	Personally, I’m mainly for requesting it. For example, the simple ankle complaint that we get as a physio. If the Ottawa Ankle rules are positive, you first have to refer the patient via the GP. I think that task can easily be done by a physiotherapist	I would rather expect an explosion in the cost of applied treatments if this is given in the hands of an ESP or an explosion in consultation time (multidisciplinary consultations)	Well that diagnostic imaging, that seems excellent to me. I think that as a physiotherapist you are very much helped if there is an image known, or a scan or something.	I think it fits very well within the scope of ESP. To bet on that. You can decide with a relatively limited amount of extra training.
Direct access	Yes, direct access. But that is more a matter of definition. I think we already do that.	X	Yes, as you said: That is already here. And I only like it as a patient that I can come and that I do not have to go to the doctor first.	Yes, fine for me. Then you also see that it does not deliver any calamities. Because actually it is already a form of triage, the screening of red flags.
Giving injections.	Yes, I think so. I think you should do that in the same way as a GP or orthopedist. You have to make a good diagnosis, take the right considerations for why you use it. Then it must be possible. But then I would also limit it to the shoulder and knee, because they are the easiest, and stay away from the other joints.	I would rather expect an explosion in the cost of applied treatments if this is given in the hands of an ESP or an explosion in consultation time (multidisciplinary consultations)	If an ESP proposes to give me an injection, I would first like to check with the doctor. I personally believe that people have to do what they are good at. And if that is what they are trained for and good at and the doctor does that once in a blue moon. Then I would certainly let that be done by the ESP.	You get so much on your neck, and why? What are you going to inject? And why do not you leave that to the professionals who are now trained for it?
Referring to specialists	You get more and more people through the direct access and then you need to send them first through the GP so they then end up in the second line. With which you take the patient away from the GP, less work pressure for the GP.	X	Yes, I think it’s fine. I do not know what the second line thinks about it? But I think it must be possible. Well, I think they should consider when they should refer. Because I think you have to prevent the specialists from saying: “Stop, we are going mad, all these physio’s that just refer. I’m already so busy.	Yes, selecting which patients go to the second line and which do not. It is on the one hand the possibility to refer, and an important task is also to limit it and prevent it from being referred.
Requesting and/or taking blood tests	But I also think that vitamin B, all kinds of other vitamins, a piece of fatigue. I would be very happy if I did not have to ask the GP every time	To do this really well and safely, extensive physiological knowledge is needed.	I do not quickly see an application for that. Perhaps I am too pragmatic, but then I would say: There are better posts for it. They are hygienic and they are all on temperature. In the context of efficiency, the hospitals do no different and are professional in it and I would say: let them continue to do it.	I think blood tests go pretty far. Sure, everything is possible, but I think it’s going pretty far.
Work capacity testing	That is a very difficult one. The GP is not able to do that either. This is often only a company doctor who can actually and legally establish this. So I have my doubts about that.	There is a great need for this, and the current GP and physio cannot judge this.	I also find this a difficult one and I wonder if patients will accept it instead of a company doctor. That is a very sensitive subject, whether people are allowed to work or what kind of work or what percentage. I don’t know if patients would accept that from someone who isn’t a doctor. I think they can do it, but only in an advising role	Yes, I find an interesting one. I think that there are opportunities. That an occupational physiotherapist may be more useful to a labor physician.
Requesting laboratory tests	But I also think that vitamin B, all kinds of other vitamins, a piece of fatigue. I would be very happy if I did not have to ask the GP every time.	To do this really well and safely, extensive physiological knowledge is needed.	Well, that also depends a bit on whether the ESP has enough know-how to make that assessment.	I do not see that at all so that an ESP should do that. I think that if there is any doubt about it, he has to go to the doctor.
Giving a medical diagnosis	Yes, if you can have additional research done and you get these things inside, then you could certainly make a medical diagnosis.	X	Yes, I also find a difficult one because you are not a medical doctor. I do not think so. I also think of an advisory role again, but do not really make a diagnosis.	No that is not possible. In the end you can never, according to me, make a medical diagnosis as long as you do not yet have the status of a medical practitioner.
Listing patients for hip or knee replacement	You can of course refer. If you have someone with obvious osteoarthritis of the knee and that is limiting their function and so on, then you can say: well, it is an idea to think about a new knee, I will send you to the orthopedist. Putting it actually on the operation list seems complicated to me. The person can use certain medication that must first be stopped for a while. The orthopedist wil probably say: I want to see that patient first before I use a knife.	Assessing whether and which surgery is required is the domain of the operator. Is it better to apply a valving osteotomy or hemiprosthesis, and which surgical technique? The operator must take into account additional issues such as urgency. All things that only the surgeon can judge.	Well, but it seems to me that the specialist would like to know what kind of patient he gets on the table and that he does not just get people from his or her hospital in all sorts of places. I do not know how that goes with responsibilities and things like that.	Yes, I think this goes pretty far too. If you are going to do that, then you do not need orthopedics. The question is whether you should want that. When you need orthopedics, they have to give that judgment. And then the orthopedic surgeon will operate. You can say: I refer to the second line.
Roles	Physiotherapist	General Practitioners	Patients	Indirect stakeholder
Working in a multi-disciplinary team	For sure. I think that we sometimes have to be a little more multi-disciplinary and also thrive very well, because other care providers depend on other care and vice versa we also depend on their care. If we were to make better use of it, the quality of the total package would be better.	In collaboration with the GP and especially specialists ideal	It seems to me, it is never wrong to have some other disciplines in a team, if you work in a health center, that you still have someone to discuss the situation.	Yes, that is perfect. If you are talking about: Someone comes in with musculoskeletal complaints and they report to a central desk. As far as I am concerned, it will not go to the GP but to the ESP who can properly assess this. You have to see it like that.
Working as a consultant	Not so much to really start a whole treatment process with the patient, but to look at it: okay, this patient is suitable for this type of physio and then goes there, or just goes there. But that you coordinate or determine that as a GP role, but no more than that.	X	Yes, it seems to me a task in itself. Provided sufficient work experience as a physiotherapist. You know what you’re talking about, I think. Seems fine	I think that is a very good one. Because I think that’s what the doctor is missing. You will be very happy with this if you do as ESP.
Having an educational role	I think that someone must have certain qualities, but in the end I have also become a teacher here. But that does not mean that every master must be able to do that.	I do not see why an ESP would be pre-eminently qualified as a teacher	Yes, but I would say: stay in practice. Because everything changes quickly, so stay up to date. Then it seems right to me to teach your colleagues.	I do not see it that way, no. In my opinion, this should not necessarily be a role for ESP. A physio can also like to do that. An ESP could do that too, but in my opinion that does not have to be a role for an ESP.
An ESP role separated from a physiotherapist role	Difficult. You will probably also work in primary care as a physiotherapist. Only it is not the intention that you as ESP will fill your own agenda or that of your colleagues. My advice will then still be to separate as good as possible.	X	That seems to me very difficult for the person concerned. To just be a physio in one moment. You always take it with you.	Yes, that depends on how technically it is regulated. If it’s a new profession, or. And otherwise you stay in the basic physiotherapist, so you can put your skills and knowledge in different places. But I do not care if you are ESP in the general practice or the physiotherapy practice.
A leadership role	Yes, especially musculoskeletal complaints. Very good though! The GP is in charge of patients with co-morbidities. This is how the care is now also organized. Maybe sometime in the future an ESP, but now it is clear the GP.	I do not think it’s useful in a medical team.	No, when I look at my own work, you have people who grow into a manager. And sometimes you do not do any work at all that you’re used to do, but you know the ropes. So yes, but you need different qualities and not every ESP could do it.	The role of case manager could well lie with the ESP in the primary care practice. Up to a certain level. Up to and including the movement-related aspect.
A role as a doctor of physiotherapy	I have a little trouble with physio-doctor. I think a doctor does a bit more than the points we just went through. So if you put yourself down as a doctor, then I wonder if that does not give a wrong picture.	X	Then they should have started studying medicine hahaha. Yes, I do not know. I also do not know how that is with such an oath and so. Of course you also have to deal with that.	I do not think so. I do not see that for me. Then you also have to get a lot of medical training. And then you go more towards the GP and the orthopedic surgeon. I do not see the added value of why someone should do that.
Working in labor related care	I do not think you should see this as an ESP role.	If independent of the own patient	I think that moving, how you sit, how you deal with stress. I also think that physical therapy can play a positive role.	Yes, you mentioned a few and I think: In principle an ESP could function here, all per specialism. Only then must he be trained more specifically.
Working in primary care arthritis care	Yes is part of it, but not as a specific role I think.	It has a more chronic and specialized character. I see ESP more as a quick and generalist, I would rather find guidance in rheumatism fit with a regular (specialized or not) physio	Yes, I’m pretty open to that. Because otherwise you have to go through a whole circle before you get help. Those roads are much shorter.	Yes, I think you should rather see it as a leading role and consultant. That the person then indeed, depending on the specialist setting, that you can refer the person to the right physiotherapists or first-line practices or health centers.
Specialized in hand therapy	Hand therapy already exists. I wonder what specifically for ESP is then.	X	Yes, I think it is possible. As I see it: the ESPs can simply specialize in certain areas and they only become more expert. So I would applaud that, I think.	Yes, I find it difficult. I am not so familiar with hand therapy myself, so now I do not know what the level of the hand therapist is. But it is true that there are some good things about it and there will also be a lot of demand for it. So that as a super specialist can also find a place.

## Abbreviations

ESP: Extended Scope Physiotherapy/Physiotherapist
GP: General Practitioner
