# Correction to: Identifying goals, roles and tasks of extended scope physiotherapy in Dutch primary care- an exploratory, qualitative multi-step study

**DOI:** 10.1186/s12913-021-06088-x

**Published:** 2021-01-29

**Authors:** Ferdinand Bastiaens, Di-Janne Barten, Cindy Veenhof

**Affiliations:** 1 Physical Therapy Sciences, Program in Clinical Health Sciences, University Medical Center Utrecht, Utrecht, The Netherlands; 2 Research Group Innovation of Human Movement Care, University of Applied Sciences Utrecht, Utrecht, Netherlands; 3 Department of Rehabilitation, Nursing Sciences and Sport, University Medical Center Utrecht, Utrecht, Netherlands

**Correction to: BMC Health Serv Res 21, 19 (2021)**

**https://doi.org/10.1186/s12913-020-05986-w**

Following the publication of the original article [1], it was noted that due to a typesetting error Figs. 1, 3 and 4 need to be updated with a new version.

**Fig. 1:**
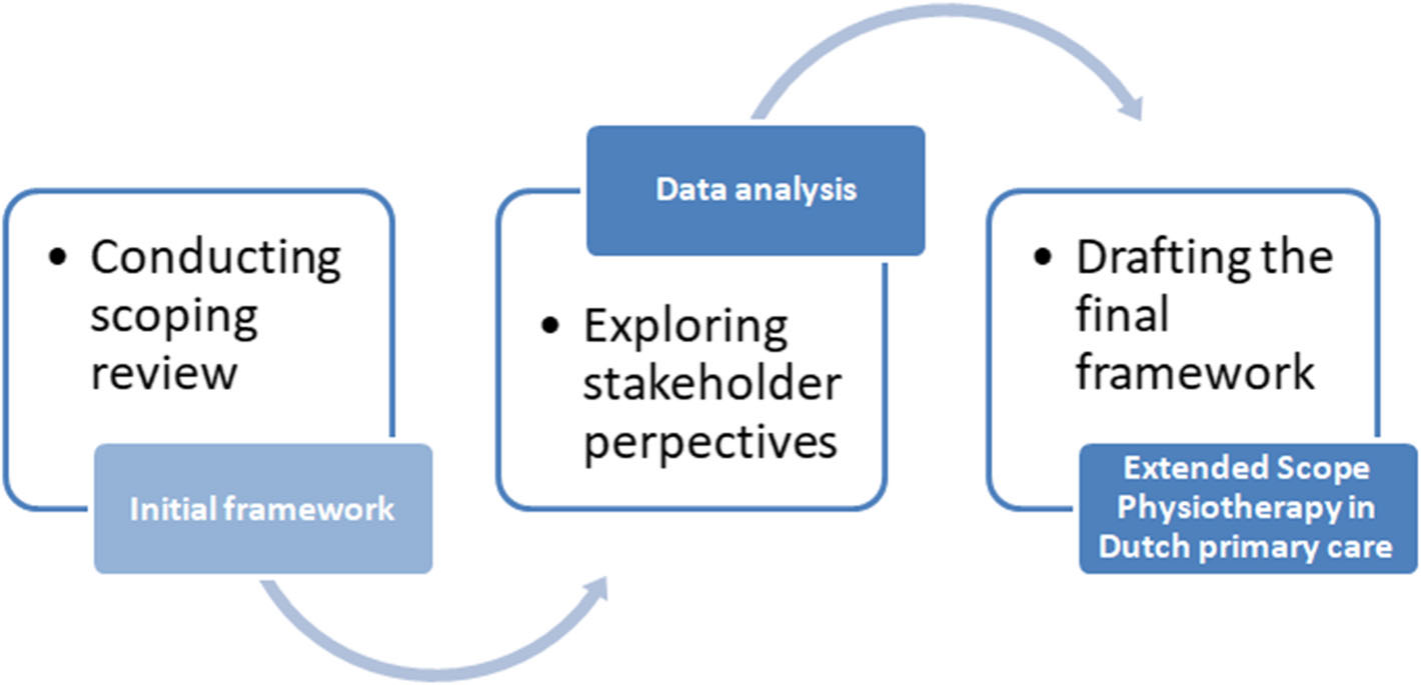
Iterative process of developing the framework of Extended Scope Physiotherapy in Dutch primary care

**Fig. 3:**
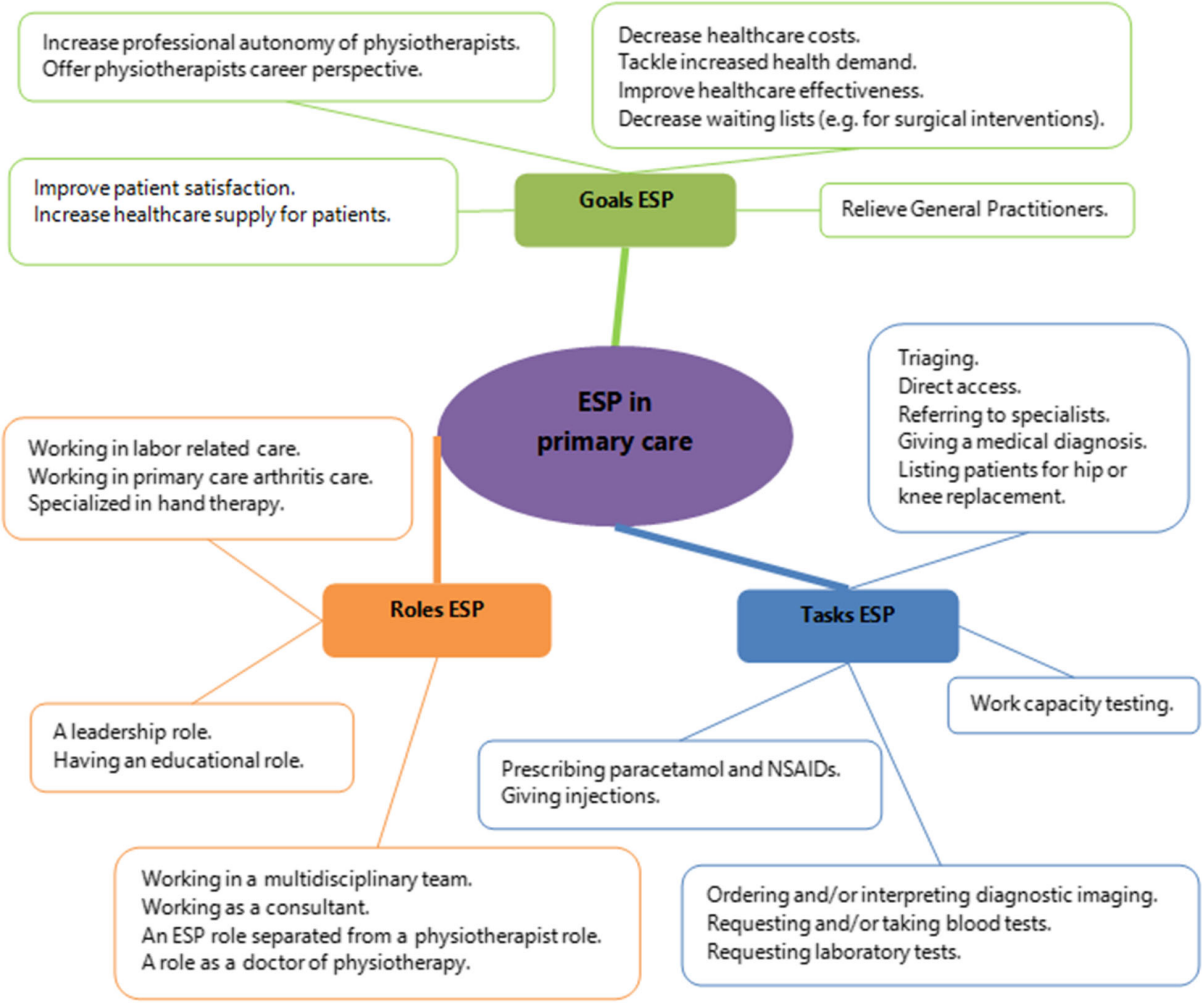
Initial framework Extended Scope Physiotherapy (ESP) in Dutch primary care

**Fig. 4:**
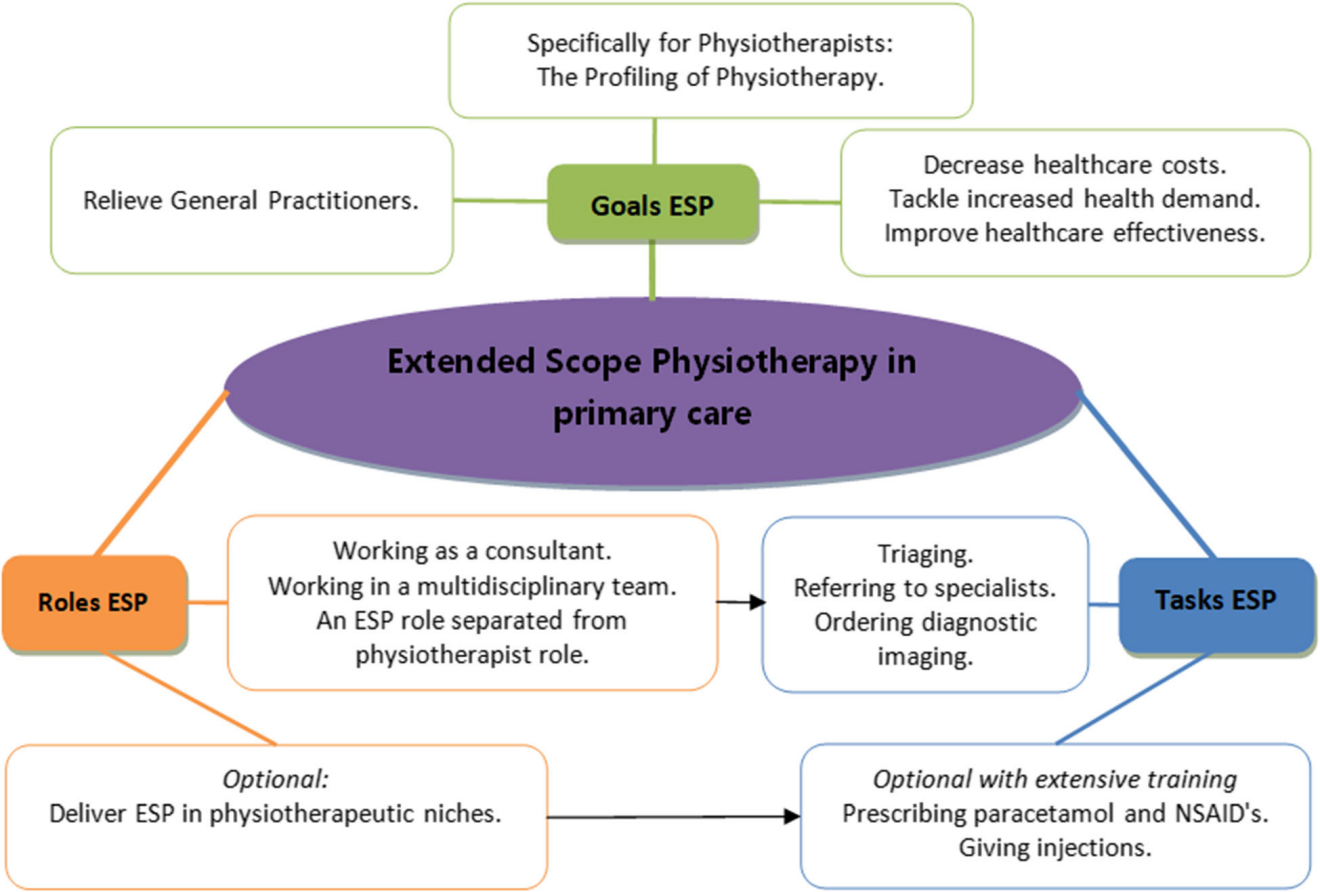
Final framework Extended Scope Physiotherapy in Dutch Primary care

The updated figures have been included in this correction, and the original article has been corrected.

## References

[1] Bastiaens F (2021). Identifying goals, roles and tasks of extended scope physiotherapy in Dutch primary care- an exploratory, qualitative multi-step study. BMC Health Serv Res.

